# REBUILDING THE NURSING HOME WORKFORCE: A TIME FOR CHANGE

**DOI:** 10.1093/geroni/igac059.1198

**Published:** 2022-12-20

**Authors:** Louisa Holaday, Jasmine Travers

**Affiliations:** Icahn School of Medicine at Mount Sinai, New York, New York, United States; New York University, New York, New York, United States

## Abstract

The COVID-19 pandemic magnified several long-standing problems in nursing homes, including issues within the nursing home workforce. Issues include staffing shortages, high turnover, low pay, inadequate training, poor treatment and limited access to resources. Research suggests that workforce issues of these kinds have the potential to significantly threaten and further increase disparities in the quality of care for nursing home residents. Thus, solutions are needed that ensures the nursing home workforce receives adequate investment so that these critical personnel are able to more effectively do the work that they do. In this presentation, workforce issues salient within the nursing home setting will be elaborated and potential solutions to mitigating these issues will be discussed.

